# Correlation between footpad lesions and systemic bacterial infections in broiler breeders

**DOI:** 10.1186/s13567-019-0657-8

**Published:** 2019-05-22

**Authors:** Ida Cecilie Naundrup Thøfner, Louise Ladefoged Poulsen, Magne Bisgaard, Henrik Christensen, Rikke Heidemann Olsen, Jens Peter Christensen

**Affiliations:** 1 Department of Veterinary and Animal Science, Faculty of Health and Medical Sciences, University of Copenhagen, Stigbøjlen 4, 1870 Frederiksberg, Denmark; 2 Bisgaard Consulting, Horsevænget 40, 4130 Viby Sjælland, Denmark

## Abstract

Footpad lesions are an important factor in evaluation of animal welfare in broilers regulated by law; however, no legal requirements have been set for the parent birds. Nevertheless, the present study confirms that foot health in broiler breeders declines significantly with increasing age, thus potentially impairing the animal welfare due to pain and discomfort from footpad dermatitis. Furthermore, this is the first report demonstrating a correlation between the presence of footpad lesions and systemic bacterial infections with Gram-positive cocci in broiler breeder birds.

## Introduction, methods, and results

In intensive poultry production systems good footpad health is crucial to obtain high levels of animal welfare and high production yields. In the meat sector, this link is poorly described in broiler breeders, whereas in the broiler industry this issue has been addressed in the minimal animal welfare regulations applied in the European Union (EU) [1–9]. It was recently documented that footpad integrity in broiler breeders declines throughout production [10]. Broiler breeders are kept in equally intensified production systems as their progeny, however, the parent birds live for a much longer period (approximately 60–64 weeks of age). This requires optimal management routines at all stages of life of the parent birds.

Foot health of broiler breeders is not mentioned in the European Directive on minimum rules for the protection of chickens kept for meat production [9], thus rendering foot health in broiler breeders in a vacuum where animal welfare is potentially compromised due to pain and discomfort from footpad dermatitis. This may not only have immediate consequences for affected birds but may also pose increased risk for getting a fatal infection due to broken epithelial lining of the footpad. Furthermore, the prevalence of infections with Gram-positive cocci is increasing during the production cycle [11]. Therefore, we hypothesize that footpad lesions may serve as a predisposing factor for this type of infections. The aim of the present study was to investigate whether footpad integrity was associated with bacterial infections in broiler breeders throughout a full production cycle.

Feet from dead broiler breeders from four Danish breeder flocks (A-D) was collected in a previous study as described in detail by Poulsen et al. [12]. Briefly, the flocks were followed throughout a full production cycle (20–60 weeks of age) and up to 10 random dead-on-farm birds weekly were collected and stored at −20 °C until post-mortem examination. All the collected birds (*n* = 997) underwent full post-mortem examination and the cause of mortality was determined [11]. Bacteriological examination was performed if observed macroscopic pathology in the bird was found to be suggestive of a bacterial infection (e.g. salpingitis, peritonitis, arthritis, septicaemia, amyloidosis, endocarditis and pododermatitis) [11].

In all houses, in all four farms, the bedding consisted of wood shavings and the bedding quality had similar appearance. The bedding quality was assessed by the ability to form a clump after manually squeezing a handful of litter. If the litter remained loose and friable after squeezing the bedding quality was regarded as dry and loose. The bedding layer consisted of loose and dry bedding material mixed with faecal matter throughout the layer, which in all farms were approximately 30–50 cm deep. No clumping of litter material was observed at any point examined (3–6 points per house) in each of the houses.

Besides the lesions associated with the death of the bird the footpad health was recorded for both feet in each bird (*n* = 924). Assessment of the feet was based on the presence of macroscopic lesions. Footpad lesions were defined as presence of hyperkeratosis ( >2 mm, minimum 3–4 affected papillae), ulcer and/or necrosis of the epidermal, dermal or subcutaneous tissue of the central footpad on each foot. No distinction between lesion severities was made. Prior to slaughter, at approximately 60 weeks of age, all four flocks were visited to assess on-farm foot health in the live birds. From each flock, both feet from 60 birds were examined visually and by palpation after brushing of litter material with a shorthaired brush. The birds were randomly selected at the same spots as for the bedding assessment. It was recorded if the bird had footpad lesions as defined for the feet of the dead birds. To evaluate whether the footpad health of the dead birds may function as a surrogate marker for footpad health in live birds, the proportion of footpad lesions in 60 weeks old live birds were compared to the proportion of footpad lesion in dead birds older than 50 weeks. For statistical analysis of the data Chi square and z-test with Bonferroni corrections for multiple comparisons between factors (e.g. flocks, age intervals), contingency tables, and Fisher’s exact test for comparing correlation between two variables (e.g. correlation between foot health and cause of mortality) were performed using GraphPad Prism, Prism 7 for Windows (GraphPad Software, La Jolla, CA, USA) and SPSS Statistics 22® (IBM Corp., Armonk, NY, USA). Relative Risk (RR), their confidence intervals (CI) and Chi square in GraphPad Prism were used for risk calculation of bacterial infection and foot health. Significance levels was set to *P* < 0.05.

There was a significant correlation between footpad lesions and overall aetiology in non-outbreak related mortality (Pearson Chi square, *P* = 0.004, data not shown), meaning that a high proportion of birds that died of infectious causes had footpad lesions, whereas this was not the case in birds that died from non-infectious causes.

There was marked flock variation in the proportion of dead birds with footpad lesions (Table 1). The proportion of dead birds with footpad lesions was significantly higher in flock A (67.3%) and D (69.1%) when compared to flock C (54.3%).

**Table 1:** **Distribution and prevalence of footpad lesions in broiler breeders dying from non-outbreak related mortality in relation to age and flock**

Overall foot health^1^	Age interval (*n* = 919)^2^				Total	Flock (*n* = 924)				Total
	20–29	30–39	40–49	50-		A	B	C	D	
No lesions										
Count	89^a^	111^b^	87^c^	54^c^	341	70^a^	82^ab^	129^b^	63^a^	344
% within age interval/flock	66.9	40.5	29.9	24.4	37.1	32.7	36.6	45.7	30.9	37.2
% of total	9.7	12.1	9.5	5.9	37.1	7.6	8.9	14.0	6.8	37.2
Lesions										
Count	44^a^	163^b^	204^c^	167^c^	578	144^a^	142^ab^	153^b^	141^a^	580
% within age interval/flock	33.1	59.5	70.1	75.6	62.9	67.3	63.4	54.3	69.1	62.8
% of total	4.8	17.7	22.2	18.2	62.9	15.6	15.4	16.6	15.3	62.8

^1^Different superscript letter denotes a subset of Age interval or Flock categories whose column proportions differ significantly from each other at the .05 level.

^2^Date of death was not registered for five birds, therefore no age could be calculated.

**Figure 1:**
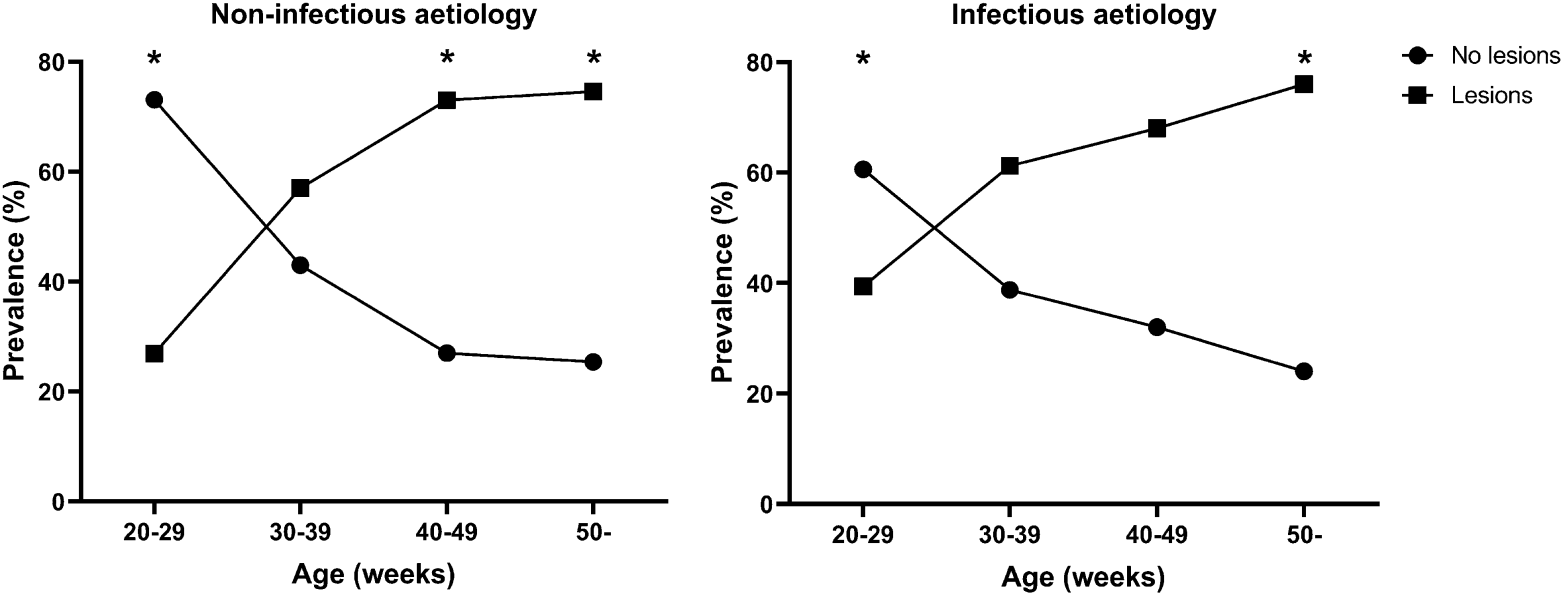
**Prevalence of footpad lesions in birds (*n* = 924) dying from non-infectious or infectious aetiology in relation to age.** * denotes significant difference in the proportions of birds with footpad lesions between age groups (*P* < 0.05, Chi square, z-test and Bonferroni correction).

The proportion of footpads without lesions decreased significantly throughout the full observation period (Table 1) regardless cause of mortality (infectious or non-infectious) (Figure 1). Healthy feet with no lesions were most commonly observed in the young birds (66.9%), whereas the proportion of birds with footpad lesions was highest in birds older than 50 weeks (Table 1). In Figure 1 it is shown that the proportion of birds with good footpad health was significantly higher in the group of young birds (20–29 weeks) regardless of the overall aetiology (*P* < 0.05). In the older birds, the proportion of birds with footpad lesions increased significantly.

Table 2 presents the correlation between footpad health and bacteriological cause of death. Typical pathology related to infections with Gram-positive bacteria were arthritis, septicaemia, amyloidosis, endocarditis and pododermatitis (data not shown). All conditions presenting pronounced inflammation, exudation, congestion, and/or amyloid deposition. The total number of birds dead from an infection with Gram-positive cocci were 126 birds. The isolated coccal species were *Staphylococcus aureus* (*n* = 76), *Enterococcus faecalis* (*n* = 29), *Staphylococcus* *agnetis* (*n* = 17), *Enterococcus* *hirae* (*n* = 2), *Staphylococcus* *lentus* (*n* = 1), and *Staphylococcus simulans* (*n* = 1). The relative risk (RR) of mortality in relation to a Gram-positive coccal infection (*n* = 126) was significantly higher in birds with footpad lesions than in birds with no footpad lesions. (RR = 1.605, CI 1.115–2.326, *P* = 0.0105). No correlation between footpad health and *Escherichia coli* infections (*n* = 317) was observed. (RR = 1.071, CI 0.8902–1.294, *P* = 0.4720).

**Table 2:** **Contingency table on the correlation between presence of footpad lesions and bacterial infection**

	Gram+ infection	No Gram+ infection	*P-*value^a^
Lesions	92	488	*P* = 0.0105
No lesions	34	310	

	*E. coli* infection	No *E. coli* infection	
Lesions	204	376	*P* = 0.4720
No lesions	113	231	

^a^Chi square

**Figure 2:**
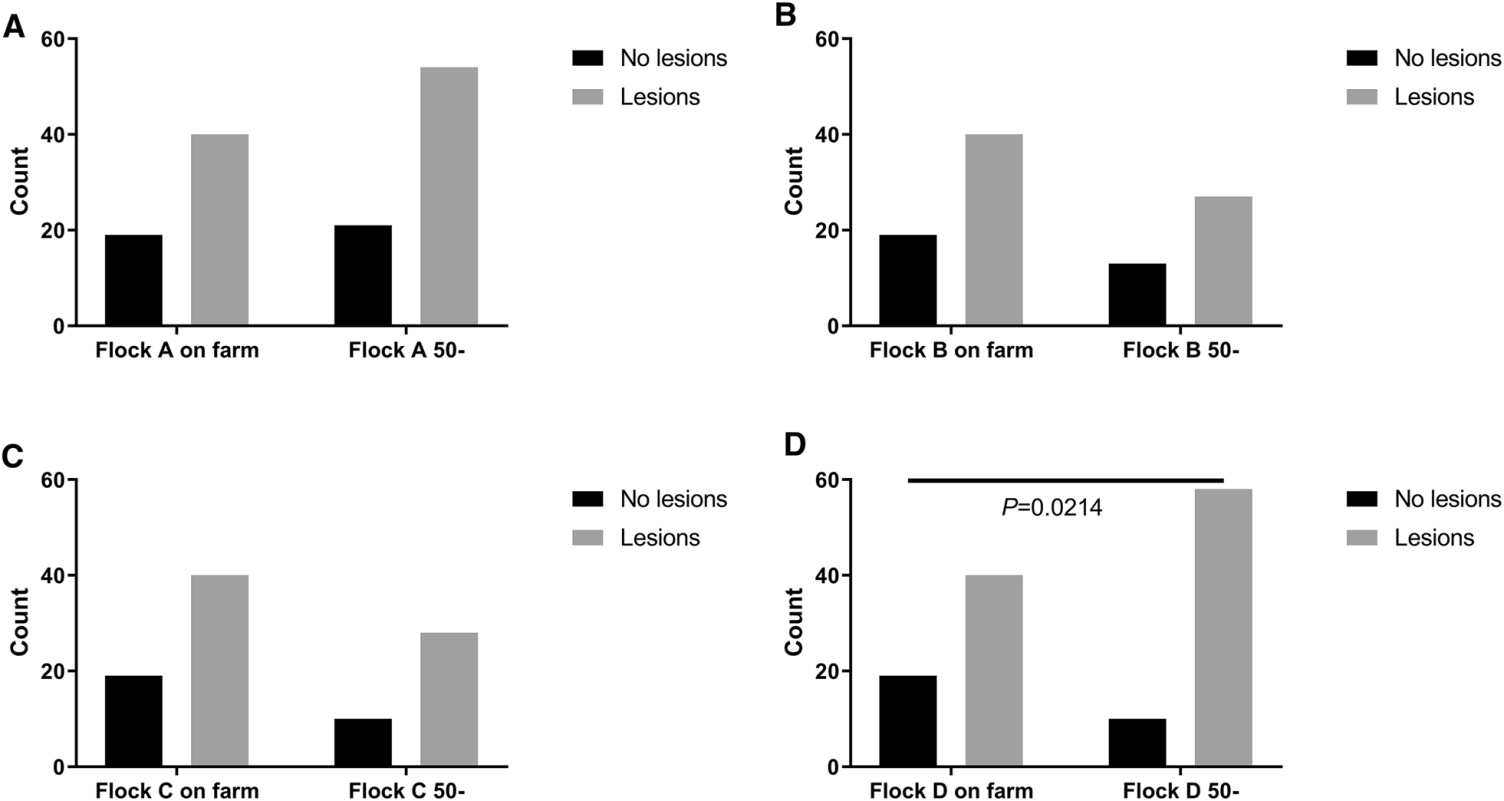
**Flockwise (A–D) comparison of the proportions of footpads with no lesions or with lesions in 60 weeks old birds on farm and in the dead birds older than 50 weeks.** Bar denotes statistical difference of the proportion of footpad lesions/no lesions within flocks (Fischer’s exact test).

Comparison of the on-farm observations of the footpad health in the live birds to the dead birds older than 50 weeks (Figure 2) revealed that the proportions of the birds with no lesion and birds with lesion did not differ within flock A, B, and C (*P* > 0.05). In flock D the dead birds older than 50 weeks (Flock D 50- weeks) had a higher proportion of dead birds with footpad lesions than observed on-farm live birds at the end of production (*P* = 0.021).

## Discussion

Footpad lesions or footpad dermatitis is a well-known factor impairing welfare in broilers [1, 2]. However, little published information concerning broiler breeders is available. A recent study in broiler breeders indicates that both the prevalence and the severity increases over time [10]. In the present study, footpad lesions were observed with increased frequency during the observation period resulting in more than 70% of the dead birds having lesions from week 40 and onwards. Furthermore, a correlation between footpad lesions and mortality due to infection with Gram-positive cocci was demonstrated. Meaning that birds have a 60.5% increased risk (RR = 1.605) of dying from a Gram-positive coccal infection when having footpad lesions compared to having intact footpads. This fits well with the observed age-related deterioration of footpad health enabling Gram-positive cocci to invade through the compromised epithelial lining. Staphylococci is often found as part of the skin flora of vertebrates, including chicken (up to 90%) [13]. Furthermore staphylococci is frequently associated with abscess formation in the central footpad (pododermatitis) due to invasion through damaged epithelium on the footpads [14, 15]. A correlation between footpad lesions and *E. coli* infection could not be established; despite *E. coli* being the most important bacterial pathogen in poultry production [16]. A possible explanation for the lack of correlation may be attributed to the most common routes of infection with *E. coli*, namely through the upper respiratory tract in young birds [17] or ascending through the reproductive tract from the cloaca in egg laying birds [18].

The comparison of the foot health of live birds at end of production to the oldest group of dead birds ( > 50 weeks old) suggests that assessment of foot health in dead birds may provide sufficiently reliable data on the foot health in a flock over a given time period provided enough birds being assessed. The observed difference in foot health in dead birds and in live birds within one flock (D) may be explained as coincidental. In contrast to broilers where footpad health is an established marker for welfare and routine assessments is demanded by law [9], the parent stock do not have routine surveillance on the foot health despite a much longer production period. This vacuum of protection of animal welfare in broiler parent stock in combination with the observations in the present study and the work done by Kaukonen et al. [10] the authors are of the opinion that routine assessment of footpad health in broiler parents is recommendable. However, the methodology needs adjustment and validation for the use in broiler breeder flocks in order to have the intended positive effect on animal welfare.

Finally, a link between systemic Gram-positive coccal infections and footpad lesion was demonstrated for the first time, thus confirming our hypothesis. The explanation for this, however, needs further investigations, but it seems likely that the damaged footpad may serve as port of entry for the cocci present in the environment.

## Data Availability

The datasets during and/or analysed during the current study available from the corresponding author on reasonable request.

## Abbreviations

CI: confidence intervals
EU: European Union
RR: relative risk
